# Osteopathy and Mental Health: An Embodied, Predictive, and Interoceptive Framework

**DOI:** 10.3389/fpsyg.2021.767005

**Published:** 2021-10-27

**Authors:** Lucas Bohlen, Robert Shaw, Francesco Cerritelli, Jorge E. Esteves

**Affiliations:** ^1^Osteopathic Research Institute, Osteopathie Schule Deutschland, Hamburg, Germany; ^2^Scandinavian College of Osteopathy, Gothenburg, Sweden; ^3^Australian Research Centre in Complementary and Integrative Medicine (ARCCIM), University of Technology Sydney, Ultimo, NSW, Australia; ^4^Clinical-based Human Research Department, Foundation COME Collaboration, Pescara, Italy; ^5^Research Department, University College of Osteopathy, London, United Kingdom; ^6^International College of Osteopathic Medicine, Malta, Italy

**Keywords:** active inference, embodied cognition, interoception, mental health, osteopathy, perceptual inference, predictive coding, touch

## Abstract

Globally, mental and musculoskeletal disorders present with high prevalence, disease burden, and comorbidity. In order to improve the quality of care for patients with persistent physical and comorbid mental health conditions, person-centered care approaches addressing psychosocial factors are currently advocated. Central to successful person-centered care is a multidisciplinary collaboration between mental health and musculoskeletal specialists underpinned by a robust therapeutic alliance. Such a collaborative approach might be found in osteopathy, which is typically utilized to treat patients with musculoskeletal disorders but may arguably also benefit mental health outcomes. However, research and practice exploring the reputed effect of osteopathy on patients with mental health problems lack a robust framework. In this hypothesis and theory article, we build upon research from embodied cognition, predictive coding, interoception, and osteopathy to propose an embodied, predictive and interoceptive framework that underpins osteopathic person-centered care for individuals with persistent physical and comorbid mental health problems. Based on the premise that, for example, chronic pain and comorbid depression are underlined by overly precise predictions or imprecise sensory information, we hypothesize that osteopathic treatment may generate strong interoceptive prediction errors that update the generative model underpinning the experience of pain and depression. Thus, physical and mental symptoms may be reduced through active and perceptual inference. We discuss how these theoretical perspectives can inform future research into osteopathy and mental health to reduce the burden of comorbid psychological factors in patients with persistent physical symptoms and support person-centered multidisciplinary care in mental health.

## Introduction

It is estimated that over one billion people are affected by mental and addictive disorders worldwide (Rehm and Shield, 2019). However, the global burden of mental disorders is likely to be underestimated due to the comorbid risk of suicide (Ferrari et al., 2014) and connectedness to other health conditions (Prince et al., 2007). An estimation taking these factors, among others, into account suggests that mental illness accounts for most years lived with disability and as many disability-adjusted life–years as cardiovascular and circulatory diseases (Vigo et al., 2016). Reflecting these alarming numbers, it has been reported that only one–third of those with a lifetime severe mental illness had been in recovery–remission for at least 12 months (Salzer et al., 2018). Hence, some consider it unlikely that psychological interventions will “*reduce the prevalence, incidence, and burden of mental illness without a major shift in intervention research and clinical practice*,” which may also encompass multidisciplinary collaborations (Kazdin and Blase, 2011). Due to the high burden and comorbidity of mental and musculoskeletal disorders (Blyth et al., 2019; GBD 2019 Diseases and Injuries Collaborators, 2020), it is critical to building multidisciplinary collaborations between mental and musculoskeletal specialists (Tyrovolas et al., 2020). Musculoskeletal disorders accounted for approximately 1.3 billion prevalent cases in 2017 (Safiri et al., 2021) and had the highest contribution to global disability (GBD 2017 Disease and Injury Incidence and Prevalence Collaborators, 2018). Taken together, musculoskeletal and mental disorders are the two main contributors to disability worldwide (Blyth et al., 2019; GBD 2019 Diseases and Injuries Collaborators, 2020).

The incontestable need to integrate physical therapy and mental health services (Attoe et al., 2018) became even more critical during the COVID-19 pandemic, with a global reduction in mental and physical wellbeing (Wilke et al., 2021). Notably, mental health conditions like anxiety and depression are commonly comorbid with chronic, often musculoskeletal, pain conditions, and vice versa (Gureje et al., 1998, 2001; Gureje, 2007, 2008; Bair et al., 2008; Holmes et al., 2012; De Heer et al., 2014; Outcalt et al., 2015; Liu et al., 2018; Amiri et al., 2020). Hence, there seems to be a bidirectional relationship between pain and psychological factors or disorders (Sørensen et al., 2019). For example, depressive symptoms are prevalent in pain sufferers, and pain symptoms are prevalent in individuals with depression (Bair et al., 2003). Furthermore, the comorbidity between pain and depression predicts worse clinical outcomes and may require simultaneous treatment (Bair et al., 2003) as it is currently unclear whether only treatment of pain or depression provides better clinical outcomes (Stubbs et al., 2017). The notion that physical and psychological disorders are frequently intertwined forms part of a recent proposal for a new disease classification termed functional somatic disorders that are “*neither purely somatic nor purely mental*” (Burton et al., 2020). Therefore, it has become common practice to integrate mental and physical healthcare services (Tegethoff et al., 2015; Kohrt et al., 2018; Daré et al., 2019; Zarean et al., 2021). Some have even proposed recognizing pain management as mental health prevention (De Heer et al., 2018).

Considering the current debate on the link between musculoskeletal and mental health disorders, we propose osteopathic care as an adjuvant therapeutic intervention to improve physical and mental health and well-being. Osteopathy is commonly and effectively used to treat musculoskeletal disorders such as back pain (Franke et al., 2014, 2015; Verhaeghe et al., 2018; Johnson and Degenhardt, 2019; Dal Farra et al., 2021). Moreover, osteopathic care was proposed to be included into chronic pain management guidelines (Franzetti et al., 2021). However, preliminary research also suggested that osteopathic interventions might benefit psychological outcomes (Williams, 2007; Williams et al., 2007; Fernández-Pérez et al., 2008). More recently, research has emphasized the effect of osteopathy on psychological and psychosocial factors in individuals with persistent pain (Edwards and Toutt, 2018; Saracutu et al., 2018), and preliminary evidence demonstrated positive outcomes with the combined use of osteopathy and psychologically informed strategies (Carnes et al., 2017; Abbey et al., 2021). Nevertheless, only a few attempts have been made to investigate the effectiveness of osteopathy on mental health disorders (Dixon et al., 2020) and develop osteopathic approaches to mental health (Liem and Neuhuber, 2020). To this end, here we propose an embodied, predictive, and interoceptive framework that aims to provide theoretical perspectives on how osteopathic care might benefit physical and, in particular, mental health. Our putative framework is informed by research from the fields of embodied cognition, predictive coding, interoception, and osteopathy.

## Embodied Cognition

Embodiment is an interdisciplinary field of research spanning disciplines like philosophy, psychology, psychiatry, and neuroscience (Fuchs and Schlimme, 2009). Theories of embodiment argue that cognition and emotion depend on embodied simulations (Veenstra et al., 2016). Thus, cognition and emotion are based upon reinstatements of perception, relating to external (exteroceptive) and internal (interoceptive) sensory states, and action, relating to (proprioceptive) motor states, which produce simulations of previous experiences in one’s self (Niedenthal, 2007; Kiefer and Trumpp, 2012). Such sensorimotor information, which is generated during our experiences within the world, may be stored in sensorimotor areas of the brain where they later can be partially re-experienced or reactivated (Niedenthal et al., 2009; Price et al., 2012) in a process called “sensorimotor simulation” (Dijkstra et al., 2014). In general, embodied cognition theories argue that the body and mind are inseparable in producing cognition (Häfner, 2013) and that all psychological processes are influenced by the body’s morphology, mental representation, and sensorimotor states and experiences (Glenberg, 2010; Pezzulo et al., 2011; Körner et al., 2016). This theoretical position indicates a strong relationship between the body and cognition (Smith et al., 2019), in which the body might both constrain and enable cognition (Woloszyn and Hohol, 2017). Therefore, embodied cognition acknowledges that not only does the mind influence the body, but the body does also influence the mind (Tschacher et al., 2017) (for putatively underlying biological mechanisms, see Renoir et al., 2013; Dum et al., 2016).

In applying the concepts of embodied cognition to our putative osteopathic framework, it is essential to critically appraisal its theoretical foundations. Crucially, we should differentiate between weaker and more robust versions of embodied cognition, where the non-neural body either contributes to or constitutes cognitive processes, respectively (Venter, 2021). Thus, embodied cognition cannot be regarded as a single viewpoint. For example, Wilson (2002) proposed six key concepts “*(1) cognition is situated; (2) cognition is time-pressured; (3) we off-load cognitive work onto the environment; (4) the environment is part of the cognitive system; (5) cognition is for action; and (6) off-line cognition is body-based.*” In contrast, Goldinger et al. (2016) argued that embodied cognition includes the following propositions: “(*1) the body influences cognitive processes; (2) cognition exists in the service of action; (3) cognition is situated in the environment; and (4) cognition may occur without internal representations.*” Notably, cognition is thought to encompass the brain, body, and environment, highlighting other cognitive resources than the brain (Wilson and Golonka, 2013). This perspective might revise our brain-centered view of cognition and consciousness by acknowledging that these processes are not limited to neural events in the brain but cut across the brain-body–world divisions and should be studied accordingly (Thompson and Varela, 2001; Kiverstein and Miller, 2015). Thereby, embodied cognition challenges mind-body reductionism and Cartesian dualism (Foglia and Wilson, 2013).

The concept of embodied cognition has been both criticized (Goldinger et al., 2016) and defended (Woloszyn and Hohol, 2017), with some arguing that the embodied and disembodied perspectives in cognitive science should be integrated to account for the existing empirical evidence (Mahon and Caramazza, 2008). Although there is increasing evidence in support of the embodiment theory (see, for example, Niedenthal, 2007; Foglia and Wilson, 2013) and seminal research experiments have been performed (see, for example, Strack et al., 1988), the research results may require an explanation in neural terms (Lakoff, 2012). The neural basis for embodiment, i.e., sensing oneself as localized within a physical body, is arguably represented in the temporoparietal junction and extrastriate body area (Arzy et al., 2006). However, correlates of embodied cognition and emotion (concerning the influence of the body on the mind) may not be identified simply at the neural level. Instead, they should be researched on multiple levels, including the brain, body, and environment (Kiverstein and Miller, 2015). Still, there is evidence from neuroimaging studies supporting the theory of embodied cognition by showing that cognition is grounded in sensorimotor experience (for grounded cognition theory, see Barsalou, 2008) which is represented in sensorimotor and likely emotional and introspective brain areas (Esopenko et al., 2012; Schaefer et al., 2014; Harpaintner et al., 2020). Harrison et al. (2010) also emphasized that emotions are embodied because they generate both peripheral–autonomic and central–neuronal responses. Furthermore, some propose a role of the mirror neuron system in embodiment processes (Price et al., 2012); however, a consensus is yet to be reached (Caramazza et al., 2014; Arévalo et al., 2015). Notably, there are different approaches and stages of commitment to the embodied nature of cognition.

Enactivism is an emerging perspective in cognitive science that is closely linked to embodied cognition. The enactivist account argues that cognition emerges from the dynamic interaction of the brain, body, and environment (Gallagher and Bower, 2014). In detail, cognition evolves as an organism acts within its environment through its embodiment; in other words, cognition is embodied action (Miyahara, 2019). From an embodied and enactive perspective, the mind is action-oriented (Kirchhoff, 2018), and cognition and emotion are rooted in the body to guide action (Zatti and Zarbo, 2015). In detail, enactivism regards cognition as *embodied* (cognition is enabled through and constrained by the non-neural body), *embedded* (cognition depends on the environmental context as the organism is situated within the environment), *enacted* (cognition is for action and depends on the interaction of the embodied organism with the environment), and *extended* (cognition extends beyond the brain and body into the environment) (Stilwell and Harman, 2019). Moreover, cognition is also considered to be ecological, i.e., cognition depends on the affordances for action (field of affordances) provided by the environment (Rietveld et al., 2018). Finally, an intriguing aspect of the enactive approach to cognition is that it largely rejects the concept of an internal representation of the world and instead emphasizes that an organism “…*does not ‘have’ a model of the world, it is the model*…” (Gallagher, 2018). Arguably, this is closely linked to a critical notion of enactivism, known as *sense making*—the evaluative interaction of an organism with its environment (De Haan, 2020a).

Embodied and enactive perspectives have been recently applied to both physical and mental health conditions. Researchers have highlighted the embodied and embedded nature of pain, being “*an action that reflects the uncertainty of body and world*” (Tabor et al., 2017). Similarly to cognition, pain is considered to be an embodied, embedded, enacted, extended, but also an emotive (affective dimensions of pain) process of sense-making through a body in an environment (Stilwell and Harman, 2019). Hence, pain may be regarded as “*an embodied response to the situation*” (Miyahara, 2019) which changes the interaction of the organism within the environment and therefore the fields of affordances—either temporarily in acute pain (“altering stance”) or persistently in chronic pain (“permeating stance”) (Coninx and Stilwell, 2021). Similarly, the importance of embodied cognition has also been highlighted for psychology (Glenberg, 2010), psychiatry (Fuchs, 2009), neuropsychology (Cardona, 2017), psychotherapy (Mende and Schmidt, 2021), and psychopathology (Fuchs and Schlimme, 2009). In detail, embodied cognition has been used to reinterpret emotional disorders (e.g., depression), considering them as arising from failed sensorimotor simulation, where previous experiences of low mood are reactivated (Gjelsvik et al., 2018). Furthermore, mental disorders have been explained as disturbances of embodiment ranging from disembodiment (feeling disconnected to/or alienated from one’s own body, e.g., in schizophrenia) to hyperembodiment (feeling conspicuous to/or hyperpresent in one’s own body; e.g., in depression) (Fuchs and Schlimme, 2009; Lape et al., 2019); which was also interpreted from a psychosocial point of view (Zatti and Zarbo, 2015). Notably, the embodiment of cognition and emotion is moderated by the individual’s sensitivity to perceiving signals from within the body (interoception) (Häfner, 2013). Thus, some have hypothesized that interoception might be regarded as the fundamental substrate of embodiment (Herbert and Pollatos, 2012). Moreover, the enactivist account has also been applied to mental health disorders, psychopathology, and psychiatry (Nielsen and Ward, 2018; De Haan, 2020a, b; Nielsen, 2020). In a nutshell, these approaches argue that mental health disorders are biased sense-making (being the embodied and embedded interaction of the organism with the environment) (De Haan, 2020b) or dysfunctional behavioral and experiential processes (impairing the adaptation of striving organisms across the brain-body-environment dimensions) (Nielsen, 2020). In summary, embodiment and enactivism emphasize the interaction of brain, body, and environment in understanding the body and mind. However, to understand the role of the brain in these processes, we must turn to another closely related field of research—predictive coding.

## Predictive Coding

Predictive coding is a theoretical framework with growing influence in the field of cognitive science (Hohwy, 2020) that is closely related to, yet partially distinct from the free-energy principle, the Bayesian brain hypothesis, and (en–)active inference (Ramstead et al., 2020).

The free-energy principle was first introduced by Friston et al. (2006) with the aim of better understanding the brain. Today, the free-energy principle is widely considered a unifying theory, aiming to explain the brain and the dynamics of all living systems (Ramstead et al., 2018). According to the free-energy principle, all living, biological, self–organizing, and adaptive systems, which can be demarcated from their surroundings (including cells, brains, humans, and even societies), resist a tendency to disorder (dispersion by random fluctuations) and try to remain in (thermodynamic) non-equilibrium steady–states by restricting themselves to a limited number of states through the minimization of *free energy* (Friston, 2009, 2019; Hipolito, 2019; Kiverstein et al., 2020; Limanowski and Friston, 2020). Herein, *free energy* is defined as the difference between a system predicted state and their actual state. Thus, minimizing *free energy* means avoiding surprise to keep within physiological bounds and the entropy of the system low (Friston, 2010). A prerequisite for this notion is that different states are separated by Markov blankets which define the boundaries of a system statistically by separating the internal from the external states (Palacios et al., 2020). However, the Markov blanket itself consists of active and sensory states: (1) active states are governed by internal states but affect external states, whereas (2) sensory states are governed by external states but affect internal states (Kirchhoff et al., 2018). *Free energy* (or prediction error) is minimized using either (1) *perception*: updating the prediction based on the sensation, or (2) *action*: changing the sensation through action to match the prediction (Seth, 2013; Holmes and Nolte, 2019).

The Bayesian brain hypothesis relates to these propositions arguing that brains make inferences (predictions) about the causes of sensations using a generative model (Friston, 2012). In detail, the brain infers (predicts) the causes of exteroceptive, interoceptive, and proprioceptive sensations using both prior beliefs and current sensory input (Edwards et al., 2012). Notably, the confidence (precision) one has in the belief (prior) or sensory input (likelihood) will determine how much perception will shift toward expectation—high precision of the prior will shift perception more toward expectation. In contrast, high likelihood precision will shift perception less toward expectation (Kuperman et al., 2020). A gap between the belief (prior) and the sensory input (likelihood) is called a prediction error (or free energy), which can update the prior based on the likelihood (Kuperman et al., 2020). In other words, a self–organizing system like the brain appears to maximize the evidence for its own existence by minimizing free energy using a (generative) model of its world (Friston, 2012). It follows that if a system is minimizing free energy, surprise, or entropy, it is arguably equivalently maximizing the evidence for its model of the world (and its own existence) by minimizing prediction error (Friston, 2012).

On the other hand, predictive coding is a framework implementing the Bayesian brain hypothesis (Friston, 2012) by applying Bayesian statistical theory to brain functioning (Tschacher et al., 2017). Experimentally, predictive coding describes neural responses and Bayesian inference behavior (Aitchison and Lengyel, 2017). In neural terms, predictive coding argues that descending (top-down) predictions are conveyed from higher cortical levels (encoded by synaptic activity) down to lower cortical levels, where they are compared to ascending (bottom-up) sensory information (Kube et al., 2020b). Notably, information only goes up the cortical hierarchy if a mismatch between the predicted and actual information occurs—i.e., *prediction error* (Williams, 2018). Therefore, top-down predictions constantly “explain away” bottom-up sensory information so that only the residual prediction errors can carry information forward in the brain (Clark, 2013; Walsh et al., 2020). It has been hypothesized that both the sensory and motor systems perform this hierarchal inference, wherein efferent descending (backward–type) projections predict sensory input and afferent ascending (forward–type) projections convey prediction errors (Adams et al., 2013). Notwithstanding this, the evidence underpinning predictive coding (concerning neurophysiological evidence) is mixed, albeit clear counterevidence is missing almost entirely (Walsh et al., 2020).

The brain can minimize prediction errors in two ways: *perceptual inference* and *active inference.* Whereas *perceptual inference* involves revising the generative model based on prediction errors transmitted up the hierarchy, in *active inference*, the agent actively acts upon the world to create the state of the world predicted by the current best generative model (Venter, 2021). Active inference is based on the assumption that the brain modifies its afferent sensory input according to prior expectations (Paulus et al., 2019). On this ground, active inference means minimizing predictive error using action by actively (re–)sampling and changing sensory input to confirm the prediction and prior belief (changing the world through action to confirm one’s own beliefs) (Friston and Frith, 2015). Therefore, the active inference model may be viewed as “self-fulfilling prophesying” (Hohwy, 2020). Equivalently, uncertainty is resolved, which maximizes model evidence and is thus self–evidencing (Kruglanski et al., 2020). Therein, active inference concerns the minimization of variational free energy or evidence bound (Friston et al., 2020) and involves perceptual, action, and learning processes but also attention dynamics (Maisto et al., 2021). In general, it outlines that living organisms tend toward creating, updating, and maintaining environmental inferences to enhance adaptation (Bouizegarene et al., 2020). However, when taking the notion of active inference under the free-energy principle seriously, active inference might be termed enactive inference because it is for action (concerns the active and selective sampling of the world through action) and cannot be regarded as equal to perceptual inference (like within the Bayesian account) because perception is considered a form of action (Ramstead et al., 2020).

Predictive coding perspectives have been applied to both physical and mental health conditions. Through this lens, symptom experience results from integrating predictions about sensory information and actual sensory information (Pezzulo et al., 2019). For example, persistent physical symptoms^1^ are regarded as “failures of inference” (Henningsen et al., 2018), characterized by overweighting of prior beliefs relative to sensory information (Edwards et al., 2012). Therefore, dysfunctional expectations become immune to disconfirming information as too much precision is afforded to prior beliefs (Kube et al., 2020a). Notably, these overly precise prior beliefs predict the symptoms that are consequently experienced (Van den Bergh et al., 2017). Similarly, patients with chronic pain may show heightened pain prediction even toward harmless sensations (Hechler et al., 2016). From a mental health perspective, individuals with mental health disorders develop suboptimal models of the world based on prior information leading to disturbed perception and belief (Teufel and Fletcher, 2020). Notably, these predictive coding perspectives have been applied to enhance our understanding of mental health disorders like depression (Kube et al., 2020b), anxiety (Paulus and Stein, 2010), post-traumatic stress disorder (Linson et al., 2020), addiction (Miller et al., 2020), psychosis (Sterzer et al., 2018), and schizophrenia (Tschacher et al., 2017); where either prior beliefs are overly precise in comparison to sensory information (Kube et al., 2020b) or vice versa (Sterzer et al., 2018). In summary, predictive coding provides a new perspective to the brain’s functioning—not as a stimulus-response machine but—as an inference machine that predicts sensory information based on prior experiences. Thus, an altered weighting of prior beliefs and sensory information may give rise to physical and mental health conditions; strikingly, conditions like chronic pain (Hechler et al., 2016) and depression (Feldman Barrett et al., 2016) seem to be mainly linked to false inferences of interoception.

## Interoception

Interoception has been defined quite diversely during the past decades (Khalsa and Lapidus, 2016). However, recent consensual views describe interoception as “*the process by which the nervous system senses, interprets, and integrates signals originating from within the body, providing a moment-by-moment mapping of the body’s internal landscape across conscious and unconscious levels*” (Khalsa et al., 2018; Chen et al., 2021). In other words, interoception is “*the sense of the physiological condition of the body*” (Craig, 2002), which seems to play a role in emotion (Critchley and Garfinkel, 2017), consciousness (Seth and Friston, 2016), behavior (Tsakiris and Critchley, 2016), social cognition (Gao et al., 2019), pain (Craig, 2003), awareness (Craig, 2009), mindfulness (Gibson, 2019), homeostasis (Feldman Barrett and Simmons, 2015), and various other domains (Ceunen et al., 2016). Furthermore, interoception provides an embodied sensory experience necessary for an adaptive interaction with the environment (Seth, 2013; Farb et al., 2015).

Bodily homeostasis is maintained via interoceptive processing involving many biological systems, i.e., the visceral, immune, and autonomic systems, using nociceptive, chemosensory and thermoregulatory functions (Khalsa et al., 2018). Furthermore, it is essential to highlight that interoception does not solely comprise afferent signaling from the body to the brain. Interoceptive information encoded in the nervous system affects perception, cognition, and behavior and leads to physical sensations expressing the psychological state (Quadt et al., 2018). Accordingly, interoception comprises a range of measurable components, such as accuracy, sensitivity, attention, detection, discrimination, and self-report (Khalsa and Lapidus, 2016). From a neuroscientific perspective, interoception involves afferent signaling processes that span neural sensors, pathways, systems, and circuits (Berntson and Khalsa, 2021). Specifically, interoceptive information is transmitted to the brain via the vagus and glossopharyngeal nerves and via viscerosensory, somatosensory, chemosensory, and lamina I spinothalamic pathways (Quadt et al., 2018). The latter pathway likely comprises projections from C-tactile afferents (Pawling et al., 2017), activated through specific touch modalities conveying interoceptive and affective information (McGlone et al., 2014). Furthermore, the neural correlations of interoception are found within the insula, anterior cingulate cortex, sensorimotor regions, and regions of the occipital, temporal, and prefrontal cortex (Stern et al., 2017). For example, during heart–focused interoceptive attentiveness, brain activity is increased within the posterior insula, right claustrum, precentral gyrus, and medial frontal gyrus (Schulz, 2016). In summary, interoception can arguably be regarded as the neural underpinning of sensing one’s own body. Therefore, it seems reasonable that dysfunctions in interoceptive processing may play a role in the development and persistence of physical and mental health disorders (Bonaz et al., 2021).

Interoception can be explored through the lens of predictive coding to understand how interoceptive processing may be involved in health conditions. More precisely, this nexus can be investigated using the active inference model applied to interoception, i.e., *interoceptive inference* (Barca and Pezzulo, 2020). At its core, interoceptive inference proposes that interoceptive experiences result from probabilistic inferences about the hidden causes of viscerosensory information—according to Bayesian principles (Seth and Tsakiris, 2018). It has been postulated that descending interoceptive predictions from the generative model enslave autonomic reflexes to maintain physiological homeostasis, while ascending interoceptive information informs and updates these predictions (Seth, 2013; Feldman Barrett and Simmons, 2015; Seth and Friston, 2016). In other words, top-down interoceptive predictions are compared with bottom-up interoceptive information, whereas the mismatch between both results in interoceptive prediction errors that are precision–weighted (Barca and Pezzulo, 2020). To minimize these interoceptive prediction errors, either the top-down interoceptive predictions are revised, or the bottom-up interoceptive information is modified to convey the prediction—the former being perceptual inference and the latter active inference (Seth, 2013). However, the precision (reliability) given to either the sensory evidence (interoceptive information) or the prior belief (interoceptive prediction) determines which one will dominate prediction error minimization (Young et al., 2019). In other words, the brain is constantly minimizing prediction error (resulting from a mismatch between predicted and actual sensory information) by adapting the generative model that underlies the prediction or by altering the actual sensory information through the action of either the sensorimotor system (active inference in response to exteroceptive stimulation) or the autonomic nervous system (active inference in response to interoceptive stimulation) (Henningsen et al., 2018). From a neuroscientific point of view, it has been argued that the insula is responsible for encoding interoceptive predictions, meaning that the insula compares top-down predictions and bottom-up sensory inputs to compute prediction errors (Allen, 2020). Arguably, the neural architecture underlying interoceptive predictions in the brain is as follows: “*prediction neurons (…) in deep layers of agranular cortex drive active inference by sending sensory predictions via projections (…) to supragranular layers of dysgranular and granular sensory cortices. Prediction-error neurons (…) in the supragranular layers of granular cortex compute the difference between the predicted and received sensory signal, and send prediction-error signals via projections (…) back to the deep layers of agranular cortical regions. Precision cells (…) tune the gain on predictions and prediction error dynamically, thereby giving these signals reduced (or, in some cases, greater) weight depending on the relative confidence in the descending predictions or the reliability of incoming sensory signals*” (Feldman Barrett and Simmons, 2015).

Intriguingly, through this lens, an emotion can be viewed as interoceptive inference (Seth et al., 2012) as emotions putatively arise from active inference of the causes of changing interoceptive (physiological) information (Seth, 2013). As such, interoceptive prediction errors are used to infer emotional states (Allen, 2020), and emotional states reflect the interoceptive precision given to prior beliefs about the consequences of action, whereas mood states represent a hyperprior over emotional states (Clark et al., 2018). Therefore, interoceptive prediction errors can, for example, be a bottom-up source of anxiety (Owens et al., 2018). More comprehensively, the theory of constructed emotion argues that an emotion concept is an embodied, whole-brain representation that is created by an internal model (informed by past experiences) to predict sensory information, infer causes, guide action, and recognize consequences for allostasis through interoception; ultimately, this prediction (after prediction error minimization) becomes a perception or an experience that categorizes the sensory event and results in an instance of emotion (Feldman Barrett, 2017). Consequently, interoceptive processes, feedback, and awareness are involved with emotional states, emotion regulation, and conscious emotional experience (Füstös et al., 2013; Price and Hooven, 2018; Volynets et al., 2020). In summary, interoception crucially contributes to emotions (Critchley and Garfinkel, 2017), and the experience of emotion and interoception even share similar patterns of brain activity involving the insular cortex (Zaki et al., 2012).

Interoception can arguably contribute to physical and mental health disorders through altered interoceptive predictions (Feldman Barrett and Simmons, 2015). It is noteworthy that people perceive a range of emotions and physical symptoms quite similarly through interoception—e.g., being afraid and having an abnormal heartbeat (Carter and Ogden, 2020). Generally, altered interoceptive processing mechanisms seem to be involved in disorders of brain-body interaction such as chronic pain, functional digestive disorders, and comorbid conditions (Bonaz et al., 2021). Therefore, physical health conditions including functional neurological disorders (Pick et al., 2020), such as functional seizures (Koreki et al., 2020), but also medically unexplained symptoms (Zacharioudakis et al., 2020), and chronic pain conditions (Di Lernia et al., 2016a) seem to be associated with deficits in interoceptive processing. Specifically, chronic pain patients have a lower interoceptive accuracy than healthy people, which correlates with symptom severity (Di Lernia et al., 2016b). Furthermore, mechanically applied stimulation of C tactile fibers (activating the interoceptive system) reduces pain severity in chronic pain patients (Di Lernia et al., 2020). Therefore, altered interoception may be involved in generating bodily symptoms both in physical and mental health conditions (Schulz et al., 2020).

Currently, interoceptive dysfunction is also believed to play an essential role in mental health disorders like anxiety and mood disorders, eating disorders, addictive disorders, and somatic symptom disorders (Khalsa et al., 2018) as well as in depressive disorders (Avery et al., 2014; Feldman Barrett et al., 2016) and autism spectrum disorders (Garfinkel et al., 2016). In particular, it has been proposed that anxiety and depression are altered interoceptive states evoked due to intrusive interoceptive predictive beliefs (Paulus and Stein, 2010). On this point, Khalsa et al. (2018) have argued that: “*the role of interoception in mental illness is that interoceptive input (i.e., posteriors) becomes increasingly decoupled from interoceptive predictions issued by the agranular visceromotor cortex (priors), leading to increased interoceptive prediction error signals*” (Khalsa et al., 2018). Specifically, two interoceptive dysfunctions typically manifest in mental illness, namely hyper-precise priors (having an unreasonably high expectation of the situation that governs interoceptive changes) and context rigidity (having difficulties to adjust this unreasonably high expectation in the face of a changing environment) (Paulus et al., 2019). Overall, there is evidence for low interoceptive precision in individuals with psychiatric disorders (anxiety, depression, eating, and substance use disorders) compared to healthy individuals, which suggests that patients fail to update their precision weighting of afferent interoceptive signals (Smith et al., 2020). However, while an atypically low interoceptive ability has been reported in patients with depression, schizophrenia, addiction, eating disorders, somatic symptom disorders, and obsessive-compulsive disorders, in turn, an atypically high interoceptive ability has been observed in patients with anxiety and panic disorders (Murphy et al., 2017). Nonetheless, it is critical to consider individual differences in interoceptive processing. Whereas one patient with a panic disorder and high interoceptive accuracy may need to adjust the precision given to inaccurate high–level interoceptive predictions (reducing worrying beliefs about real but harmless sensations), another patient with a panic disorder and low interoceptive accuracy may need to adjust the precision given to inaccurate low–level interoceptive information (reducing illusory sensations that maintain worrying beliefs) (Ainley et al., 2016). Lastly, both physical and mental health disorders may benefit from interoceptive exposure therapy to facilitate interoceptive awareness. Treatment interventions using interoception seem to alleviate symptoms of psychiatric disorders like anxiety disorders, eating disorders, psychosomatic disorders, and addictive disorders (Khoury et al., 2018) while also reducing fear of pain in pediatric patients with chronic pain (Flack et al., 2018) and pain and negative affect in children with functional abdominal pain (Zucker et al., 2017).

In summary, there is growing evidence linking interoceptive deficits to physical and mental health conditions. Moreover, there is developing evidence that treatment modalities using interoceptive interventions are clinically effective—both of which may be explained from a predictive coding standpoint. However, current interventions targeting interoceptive dysfunction primarily apply behavioral (comprising meditation and cognitive behavioral therapy), pharmacological (blocking ghrelin receptors), and neural stimulation approaches (transcranial magnetic and direct current stimulation) (Chen et al., 2021). Here, we propose osteopathy as an adjuvant non-invasive, body- and touch-based approach to putatively modify interoceptive states.

## Osteopathy

Osteopathy is a form of health care that uses manual diagnosis and treatment alongside patient management approaches to optimize, restore, or maintain patients’ structure, function and well–being (Vaucher et al., 2018; Zegarra-Parodi et al., 2021). Osteopathic evaluation and treatment rely heavily on perceptual judgments regarding the nature of the patient’s problem. Therefore, it is important to distinguish between bottom-up and top-down mechanisms—relating to ascending and descending dynamics between peripheral tissues and the brain (Liem and Neuhuber, 2020). On the one hand, osteopathic hands-on approaches encompass diagnostic tests primarily using palpation and therapeutic techniques based on touch and manipulation to influence patients’ peripheral tissues. On the other hand, osteopathic hands-off approaches involve patient management procedures like patient education, psychological support, lifestyle advice, and self-management solutions to influence patients’ cognition and psychological state (Fryer, 2017a, b; Vaucher et al., 2018). Arguably, these hands-on and hands-off approaches act on peripheral tissues and the brain, respectively. Nonetheless, both involve top-down and bottom-up dynamics. For example, hands-on approaches may produce unexpected sensory input to peripheral tissues that is processed bottom-up (likelihood), however, sensory input is dependent on the brain’s expectation about the effect of hands-on approaches on peripheral tissues which is issued top-down (prior). In contrast, hands-off approaches may foster or challenge the brain’s internal model generating the expectations about, for example, sensory input from peripheral tissues which is processed top-down or bottom-up, respectively.

From an osteopathic hands-on perspective, touch and manipulative techniques are used to diagnose and treat somatic dysfunctions in the body (Cerritelli et al., 2015; D’Alessandro et al., 2016; Giusti, 2017). However, the concept of somatic dysfunction has been critically debated within the profession (Esteves et al., 2020), mainly because of the (1) unclear pathophysiology and poor diagnostic reliability (Fryer, 2016), (2) unestablished relation to health status (Moran, 2016), and (3) continuous changes in terminology and explanation (Liem, 2016; Bergna et al., 2020). While some propose a neuro–fasciagenic perspective to somatic dysfunction (Tozzi, 2015a, b), others emphasized the relation between palpatory findings and allostatic load (Lunghi et al., 2020) or movement variability (Bergna et al., 2020). Apart from ongoing work on the conceptual basis of the somatic dysfunction framework, more recently, special attention has been given to the effects of touch in general (Manzotti et al., 2020; Baroni et al., 2021) and the underlying neurological mechanisms of osteopathic care (D’Alessandro et al., 2016; Pelletier et al., 2018; Gyer et al., 2019).

Osteopathic practice is heavily influenced by models of care that inform hands-on osteopathic diagnosis and treatment. In particular, osteopathic clinical reasoning is governed by osteopathic models including the (1) biomechanical or structural, (2) neurological, (3) metabolic, metabolic–energetic, or nutritional, (4) respiratory–circulatory, and (5) biopsychosocial or behavioral model (Grace et al., 2016; Lunghi et al., 2016; Lunghi and Fusco, 2017; Sciomachen et al., 2018; Seffinger et al., 2018; Esteves et al., 2020)—all putatively underlined by a connective tissue-fascial meta-model (Tozzi, 2015a, b). These structure-function models are used in combination to assess the relevance of somatic dysfunction (or palpatory findings, respectively), prioritize treatment approaches, and guide diagnosis and treatment (Grace et al., 2016; Tamburella et al., 2019). However, the usefulness and plausibility of these models have been recently critically debated within the profession (Alvarez et al., 2020; Bettelli et al., 2020; Esteves et al., 2020; Lunghi and Liem, 2020; Ménard et al., 2020; Nesi, 2020; Sampath and Fairs, 2020; Santiago et al., 2020; Steel et al., 2020a).

Osteopathic practice is not merely defined by the sole application of single osteopathic techniques but rather the expression of its philosophy in the clinical context (Paulus, 2013). However, this osteopathic philosophy is not an epistemology *per se*, but rather comprises the following guiding principles: “(*1) the human being is a dynamic functional unit, whose state of health is influenced by body, mind and spirit; (2) the body possesses self–regulatory mechanisms and is naturally self–healing; and (3) structure and function are interrelated at all levels of the human body*” (World Health Organization [WHO], 2010). Osteopaths aim to find health instead of disease (Still, 1899) and treat the cause rather than the symptoms, even if both are distant (Paulus, 2013). Over the years, the osteopathic principles (Special Committee on Osteopathic Principles and Osteopathic Technique, 1953; Rogers et al., 2002; Giusti, 2017) have been refined (Stark, 2013) and extended (Paulus, 2013). Moreover, how osteopathic principles inform clinical practice is regarded by some authors as a defining (yet updatable) characteristic of osteopathy (Cotton, 2013; McChesney, 2013), while others criticize their missing scientific evaluation (Thomson et al., 2011; Evans, 2013) and lack of distinctiveness, plausibility, precision, and manual focus (Tyreman, 2013). In general, osteopathy is considered a person-centered (Fahlgren et al., 2015; Tyreman, 2018) and holistic (Turner and Holroyd, 2016) health care approach. This viewpoint is endorsed by patients’ perception (Lam et al., 2019) but may still not sufficiently differentiate osteopathy from other health care professions (Thomson et al., 2013). Furthermore, it is currently unclear how the nature and principles of osteopathy inform patient management strategies commonly used in clinical practice; in particular, patient/pain education and psychological support (Fitzgerald et al., 2020), lifestyle advice emphasizing diet, nutrition, physical activity, and exercise (Fleischmann et al., 2020), and self-management solutions (Vaughan et al., 2020). In summary, osteopathy arguably combines hands-on manual approaches (using touch and manipulation) informed by osteopathic models of care with hands-off patient management approaches (using patient education, psychological support, lifestyle advice, and self-management solutions) informed by osteopathic principles, both utilizing top-down and bottom-up dynamics between peripheral tissues and the brain. Ultimately, one can argue that osteopathy may enhance the patient’s knowledge and perception of health (Bohlen et Witte, personal communication, July 15, 2019).

Although osteopathic treatment mechanisms are not yet fully understood, proposals made have mainly focused on the nervous and fascial systems—these strongly link osteopathy to interoception. From a neurological perspective, osteopathy is hypothesized to follow an “interoceptive paradigm” (D’Alessandro et al., 2016). Based on previous findings showing that osteopathic treatment produces anti-inflammatory and hyper–parasympathetic effects, the authors suggested that the treatment of peripheral tissues may modify sensitization states and change interoceptive processes, thus reducing an underlying inflammatory condition. From a fascial perspective, research demonstrates that osteopathic techniques produce biological (*in vitro* and *in vivo*) effects on the fascia (Bove and Chapelle, 2012; Tozzi et al., 2012; Zein-Hammoud and Standley, 2015; Parravicini and Bergna, 2017). Importantly, the fascia seems to be an organ of interoception—80% of the afferent nerves in musculoskeletal tissues are interstitial muscle receptors located in fascial tissues, of which 90% stimulate afferent C–fibers that project to the insular cortex (Schleip and Jäger, 2012). Considering the neurological and fascial theoretical positions together, Bordoni and Marelli (2017) have argued that manual treatment of the myofascial continuum activates the interoceptive system and thus also stimulates areas of the brain that are concerned with emotions.

Initial research investigating the effect of osteopathy on interoceptive measures was heterogeneous, showing that (1) deep touch and osteopathic mobilization significantly increased interoceptive accuracy (Edwards et al., 2018); (2) myofascial release techniques increased interoceptive sensitivity, but not significantly (Cathcart et al., 2019); and (3) high velocity, low amplitude manipulation techniques did not significantly change interoceptive accuracy scores (Griffiths et al., 2019). However, a recent fMRI study by Cerritelli et al. (2020b) demonstrated that osteopathic treatment increases the interoceptive accuracy of patients with chronic low back pain and has an effect on the patients’ brain correlates of interoception by significantly decreasing the BOLD response of the bilateral insula, anterior cingulate cortex, right middle frontal gyrus, and left striatum. There might be grounds to hypothesize that these effects could be the product of activating C–tactile afferents (unmyelinated low threshold mechanosensitive C–fibers) through gentle, slow-moving touch (McGlone et al., 2017). Afferent C–tactile fibers are stimulated during affective, low force, dynamic touch and seem to activate the posterior insular cortex and reduce autonomic arousal (arguably being an interoceptive modality) (Manzotti et al., 2019). Moreover, touch stimulating these fibers may play a role in body awareness, homeostatic regulation, allostasis, emotion, and affective disorders (Crucianelli and Filippetti, 2020; Burleson and Quigley, 2021). Depression, for instance, is associated with impaired interoceptive accuracy, and massage therapy has therefore been proposed to produce its alleviating effects through the stimulation of C tactile afferents, which allegedly restores the impaired interoceptive function (Eggart et al., 2019).

Although touch plays a central role in osteopathic care, its effects on the patient go beyond tactile sensory stimulation. Research demonstrates that the cognitive status of the osteopath (focusing attention on touch vs. audition) influences the subject’s functional connectivity patterns involving brain correlates that process the interoceptive and attentional value of touch (the insula, posterior cingulate cortex, and right inferior–frontal gyrus) (Cerritelli et al., 2017a). Furthermore, recent reviews of neuroimaging studies demonstrate functional convergence for mindfulness and touch at the interoceptive cortex—this provides a rationale for investigating the combination of top-down mindfulness–informed and bottom-up touch-based approaches in the treatment of body-mind disorders that involve interoceptive deficits (Casals-Gutiérrez and Abbey, 2020; Baroni et al., 2021), including chronic pain (Di Lernia et al., 2016b) and depression (Smith et al., 2021). In general, it has been shown that implementing an audio-guided mindfulness-based practice for patients in the waiting room before receiving osteopathic manipulative treatment enhances treatment satisfaction and the patient’s feeling of safety and mindful connection to their own bodies (Hanley et al., 2021). More specifically, combining touch- and mindfulness-based interventions (from osteopathy and acceptance and commitment therapy) was reported to be feasible and beneficial in the treatment of persistent (musculoskeletal) pain (Carnes et al., 2017; Abbey et al., 2021). In summary, osteopathic treatment may influence interoceptive processing, which could be relevant to physical and mental health conditions.

Osteopaths are typically involved in the care of individuals presenting with a range of clinical conditions (Sciomachen et al., 2018), but most frequently those presenting with musculoskeletal disorders (Johnson and Degenhardt, 2019). To date, there is a growing but still limited^2^ body of evidence supporting the efficacy and effectiveness of osteopathic treatment in chronic pain conditions (Haller et al., 2020; Licciardone et al., 2020; Rehman et al., 2020; Franzetti et al., 2021) in particular musculoskeletal disorders like back pain (Licciardone et al., 2005; Franke et al., 2014, 2015, 2017; Verhaeghe et al., 2018; Switters et al., 2019; Dal Farra et al., 2021); however, some counterevidence is available as well (Posadzki and Ernst, 2011; Orrock and Myers, 2013; Nguyen et al., 2021). Furthermore, there is sparse evidence suggesting that osteopathic treatment may benefit other physical health conditions (Müller et al., 2014; Cicchitti et al., 2015; Cerritelli et al., 2016, 2017b; Ruffini et al., 2016; Lanaro et al., 2017). Hence, osteopathy is primarily used to treat physical (musculoskeletal) disorders but is also linked to mental health. From a historical point of view, osteopathy, unlike medicine (Gendle, 2016) and psychiatry (Thibaut, 2018), mainly opposed the dualistic view of body and mind and instead emphasized the unity and interaction of body, mind, and soul (Zegarra-Parodi et al., 2019b, 2021). Arguably, this set the tone for osteopathy to become a whole-person approach to health care (Wilson, 2017) that acknowledges biological, psychological, social, religious, and spiritual factors (Zegarra-Parodi et al., 2019a). Hence, it might come as no surprise that the founder of osteopathy, Andrew Taylor Still, promoted the development of an osteopathic psychiatry speciality (note that in the US osteopaths are physicians) (Bradley et al., 2003). While some have discussed the role of psychology in osteopathic care for patients with pain (Pincus, 2006), others have gone further and proposed ideas on how to work toward osteopathic psychiatry (McLaren, 2010). Despite this, little effort has been made to provide an underlying framework and implement an approach. Also, it seems somewhat questionable to develop osteopathic psychiatry due to regulatory differences between countries and the presumed incompetence of osteopaths to manage mental disorders. Hence, it might be best to discuss (1) a psychologically informed osteopathic practice addressing comorbid psychological factors in patients with physical disorders and (2) a collaborative treatment approach combining mind-based (psychotherapy) and adjuvant body-based (osteopathy) approaches in the treatment of mental disorders.^3^ Regarding this, preliminary evidence emerged that might revive discussions about the use of osteopathy in mental health as some research pointed out that osteopathic interventions might benefit psychological outcomes (Williams, 2007; Williams et al., 2007; Fernández-Pérez et al., 2008) especially in chronic pain patients (Edwards and Toutt, 2018; Saracutu et al., 2018). Further, initial research has also been conducted to investigate osteopathy’s effect on mental disorders (Dixon et al., 2020), and the first osteopathic approaches have been developed to improve mental health (Liem and Neuhuber, 2020).

**FIGURE 1:**
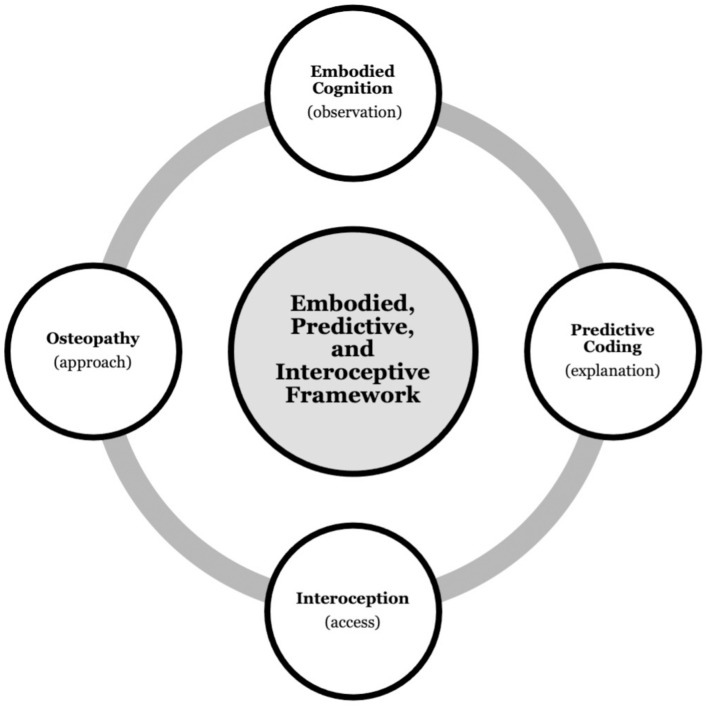
Background synthesis. Integrating the research fields of embodied cognition, predictive coding, interoception, and osteopathy to propose an embodied, predictive, and interoceptive framework to osteopathy and mental health.

Taken together, the research reviewed so far provides the foundation on which we build our theoretical framework. Simplified, embodied cognition highlights the role of the body in cognition and mental health, which is underpinned by processes elucidated in predictive coding. Further, interoception may provide an access point to influence these processes, putatively using osteopathy (Figure 1).

## An Embodied, Predictive, and Interoceptive Framework

*“If the body is the nexus by which therapies can directly alter interoceptive states, then it follows that body-based therapies should provide a more direct entry point by which to manipulate the interoceptive system and correct somatic error” (Paulus et al., 2019).*

*“From a predictive coding framework, body-focused contemplative practices may alter interoceptive processing by shifting regulatory habits from active to perceptual inference, increasing bottom–up integration of what is happening in the body rather than attempting to alter body sensation to fit top–down expectations of what should happen in the body” (Farb et al., 2015).*

Herein, we propose an embodied, predictive, and interoceptive framework to osteopathy and mental health. In the following, we firstly outline physical and mental health conditions through the lens of predictive coding and active inference, and secondly integrate these perspectives to propose a framework that aims to provide a theoretical grounding for the putative effect of osteopathic treatment on persistent physical and comorbid mental symptoms/conditions, illustrated using the example of chronic pain and comorbid depression.

On the one hand, physical symptom perception may result from altered precision weighing between interoceptive predictions (prior) and interoceptive sensations (likelihood), where either too much precision is afforded to the prior or too little precision is afforded to the likelihood (Pezzulo et al., 2019). Van den Bergh et al. (2017) have argued that the perception of physical symptoms starts with the formation of a prior (predicting the presence of symptoms based on past experiences), which is then compared to the likelihood (comprising actual afferent sensory information). Subsequently, prediction errors are minimized (if prior and likelihood mismatch), thereby generating a symptom experience (posterior) that matches both prior and prediction error (Van den Bergh et al., 2017). In the case of persistent physical symptoms, dysfunctional expectations are likely to become immune to disconfirming information, and therefore patients attribute severe pathology to benign bodily sensations (Kube et al., 2020a). In other words, too much precision is afforded to prior beliefs/predictions (Van den Bergh et al., 2017). Persistent physical symptoms can therefore be regarded as “failures of inference” (Henningsen et al., 2018), characterized by overweighting of prior beliefs relative to sensory information (Edwards et al., 2012), and these overly precise priors predict the symptoms that are consequently experienced (Van den Bergh et al., 2017).

Similarly, patients with chronic pain are believed to display a heightened prediction of pain that leads them to infer pain as the likely cause of even harmless interoceptive input due to past experiences (Hechler et al., 2016). Therefore, even if actual interoceptive information is non-noxious, chronic pain patients predict the interoceptive information to be the source of symptoms and minimize the resultant mismatch by shifting attention away from actual input (disregarding non-noxious sensations) or by making the prediction of pain come true through active inference (inferring noxious sensations)—this leads to the perception of pain through perceptual inference (Hechler et al., 2016). Reflecting these theories, patients suffering from persistent physical symptoms and chronic pain show deficits in interoceptive processing and have lower interoceptive accuracy (Di Lernia et al., 2016a, b). In summary, persistent physical symptoms and chronic pain may result from too much precision being afforded to prior interoceptive predictions that are not updated based on actual interoceptive information but confirmed through active inference processes, resulting in the perception of expected pain through perceptual inference. However, in turn, medical interventions may prompt patients to infer small interoceptive changes as a result of healing, thus leading to symptom relief through active inference without restoring bodily function (Ongaro and Kaptchuk, 2019).

On the other hand, mental health symptoms may result from an altered precision control leading to a failure of balancing prior and likelihood (Friston, 2017). In detail, heightened precision is afforded to prior predictions, thus producing prediction errors and allostatic load that contributes to, for example, anxiety and depression (Paulus et al., 2019). Therefore, mental health symptoms such as depression represent altered interoceptive states that evolve due to “noisy” interoceptive predictions (Paulus and Stein, 2010). In other words, interoceptive input seems decoupled from interoceptive predictions, thus leading to increased interoceptive prediction errors (Khalsa et al., 2018). This mismatch between predicted and sensed interoceptive information may lead to interoceptive dysfunctions such as hyper-precise priors, i.e., having an unreasonably high expectation of the situation that governs interoceptive changes, and context rigidity, i.e., having difficulties to adjust this unreasonably high expectation in the face of a changing environment (Paulus et al., 2019). Furthermore, different mental health disorders seem to be associated with either overly precise prior beliefs relative to sensory information (e.g., in depression) (Kube et al., 2020b) or vice versa (e.g., in schizophrenia) (Sterzer et al., 2018).

Interestingly, subjects with mental health disorders show low sensory precision compared to healthy individuals, possibly due to a failure to update the precision weighting of afferent interoceptive signals, suggesting that overly precise priors and imprecise likelihoods may underlie psychopathology (Smith et al., 2020). However, while many mental health disorders are linked to an atypically low interoceptive ability, others are associated with an atypically high interoceptive ability (Murphy et al., 2017). Furthermore, people with the same mental health disorder may show individual differences in interoceptive accuracy. For example, one patient may show worrying beliefs about real but harmless sensations (high precision for inaccurate interoceptive predictions), while another patient may show illusory sensations that maintain worrying beliefs (high precision for inaccurate interoceptive information) (Ainley et al., 2016). More specifically, in the case of depression, too much precision is afforded to negative prior beliefs that are not updated when confronted with disconfirming information (Kube et al., 2020b). Therefore, in patients suffering from depression, afferent interoceptive information may become decoupled from interoceptive predictions leading to noisy prediction errors that are minimized by either maintaining the predictions and not attending to the sensory information or by engaging the autonomic, metabolic and immune systems to generate the predicted sensory information (Feldman Barrett and Simmons, 2015). Arguably, this process of reducing interoceptive prediction errors limits activity and energy expenditure, thereby resulting in depressive symptoms (Feldman Barrett and Simmons, 2015).

Taken together, both physical and mental health symptoms may result from altered precision weighing between interoceptive predictions and prediction errors where either too much precision is afforded to the prior (overly precise predictions) or too little to the likelihood (imprecise prediction errors). Chronic pain and depression seem to be mainly linked to the overweighting of priors. Arguably, these beliefs predict painful and depressive states even if the actual interoceptive input is harmless and make these states come true through active inference (expected symptoms are generated through action to confirm the prediction). In order to update these beliefs and reduce active inference of pain and depression, surprising interoceptive input may be provided to increase the weight of the likelihood and generate prediction errors that can revise the belief issuing the predictions, thus, fostering perceptual inference (actual information is used to update the prediction, and perception, of symptoms).

Hence, our putative embodied, predictive, and interoceptive framework to osteopathy and mental health rests on the assumption that patients with physical and comorbid mental health symptoms (illustrated using the example of chronic pain and comorbid depression) display altered precision weighing between interoceptive predictions (expected physiological body state) and interoceptive input (sensed physiological body state). Therefore, a mismatch between both results in interoceptive prediction errors that are minimized using active and perceptual inference processes. Arguably, chronic pain and depression result from overly precise interoceptive predictions or imprecise interoceptive prediction errors; either the precision given to the prior is too high, or the precision given to the likelihood is too low. In that, patients may predict and infer pain and depression as the likely causes of uncertain but often benign interoceptive information while not attending to interoceptive prediction errors that would be able to update the maladaptive^4^ belief generating the prediction of pain and depression. Consequently, when chronic pain and depression are present, predicting allostasis (interoceptive predictions) and sensing interoception (interoceptive prediction errors) are likely underlined by inadequate certainty (precision weighting).

**FIGURE 2:**
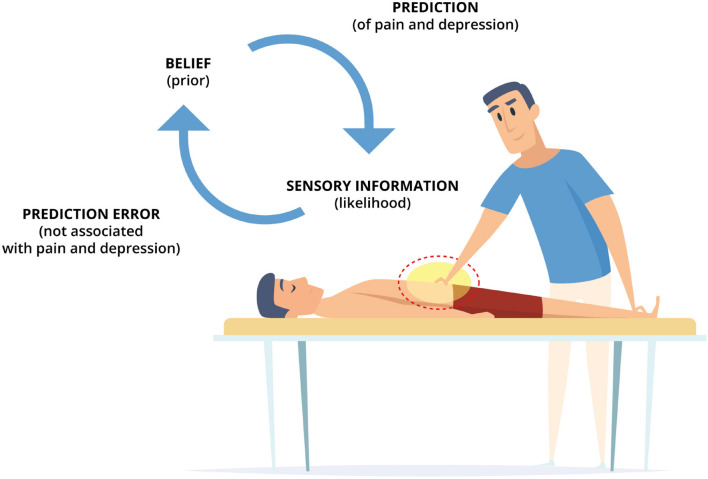
An embodied, predictive, and interoceptive framework to osteopathy and mental health. A patient with chronic pain and comorbid depression is lying in supine position, while the therapist applies osteopathic treatment strategies (e.g., providing touch-based interventions to the symptomatic bodily area). Arguably, the belief (prior) of the patient predicts physical and mental states associated with pain and depression to be the likely causes of uncertain sensory information (likelihood). However, if the provided treatment (sensory input) is not linked to these physical and mental states, this surprising mismatch between expected and actual interoceptive information generates interoceptive prediction errors. These interoceptive prediction errors are subsequently minimized using active and perceptual inference processes. If, in chronic pain and depression, high precision is afforded to the belief (prior) and low precision is afforded to the sensory information (likelihood), active inference processes are engaged which produce symptoms resembling the predicted physical and mental states associated with pain and depression through autonomic nervous system activity. However, in a healthcare setting, this might not sufficiently reduce and explain interoceptive prediction errors. Consequently, perceptual inference processes are engaged to update the prior (belief) based on the likelihood (sensory information) thus revising the generative model holding the belief and issuing the prediction. These processes, arguably, underpin osteopathic treatment and putatively reduce (the belief about and prediction of) physical and mental states associated with pain and depression by updating persistent and noisy interoceptive prediction errors (which maintain symptoms through active inference) with surprising and precise interoceptive prediction errors (which alleviate symptoms through perceptual inference).

We hypothesize that both interoceptive deficits (overly precise interoceptive predictions and imprecise interoceptive prediction errors) may benefit from the interoceptive input provided within osteopathic treatment to update the maladaptive prediction and improve the ability to attend to interoceptive information. From this perspective, clinicians should generate uncertain and surprising interoceptive input through osteopathic treatment strategies that are likely to increase the weight of interoceptive prediction errors. Osteopaths typically (but not exclusively) treat the bodily area that is linked to the patient’s symptoms of, for example, chronic pain and comorbid depression. Therefore, the patient is likely to expect the sensory information from this bodily area to evoke physical and mental states associated with pain and depression. However, if the interoceptive information produced by the osteopath is uncertain and surprising to the patient (not linked to these physical and mental states), strategies must be used to minimize this mismatch between expected and actual interoceptive information. We argue that, first, active inference processes are implemented to explain away the interoceptive prediction errors. Therein, action is used to bring in line perception with the prediction (high precision prior). In that, the autonomic nervous system is engaged to produce symptoms resembling the predicted physical and mental states related to pain and depression, because they are inferred to be the most likely causes of uncertain interoceptive input. However, the sensory input provided by the osteopath is applied in a healthcare setting (exteroceptive input) using healthcare interventions (interoceptive input), both of which are typically associated with, and should predict, health promotion (contextual factors). Thus, interoceptive prediction errors arguably gain precision (certainty), and active inference processes that produce (or resemble) the physical and mental states linked to pain and depression might not be adequate to explain away the mismatch between predicted and sensed interoceptive input or interoceptive prediction errors, respectively. Arguably, perceptual inference processes are implemented to update the prediction and underlying belief on the basis of interoceptive prediction errors. Perception is used to bring in line action and update the prediction (high precision likelihood). In that, the generative model holding the maladaptive beliefs, and issuing the predictions, may be updated based on actual interoceptive information while simultaneously improving the ability to attend to interoceptive input. Arguably, this reduces (the belief about and prediction of) physical and mental states associated with pain and depression; presumably, persistent and noisy interoceptive prediction errors are replaced with surprising and precise interoceptive prediction errors (Figure 2).

We have proposed that osteopathic treatment may facilitate perceptual inference processes by increasing the weight (precision) of interoceptive prediction errors to update the belief issuing maladaptive predictions and thus reduce physical and mental health symptoms sustained through active inference processes. This perspective is in line with the proposal that body-focused and contemplative therapies “*may alter interoceptive processing by shifting regulatory habits from active to perceptual inference*” (Farb et al., 2015). Hence, osteopathic interventions may theoretically reduce interoceptive processing dysfunction, autonomic activity, and allostatic load by increasing the precision of actual interoceptive information (likelihood), generating prediction errors that update the belief (prior), thus decreasing the mismatch between expected and actual interoceptive states. These assumptions are in line with research pointing out that osteopathic palpatory findings may be linked to allostatic load (Lunghi et al., 2020), that osteopathic treatment may reduce allostatic load (Nuño et al., 2019a, b), and that osteopathic treatment may change interoceptive processes (D’Alessandro et al., 2016), increase interoceptive accuracy, and modify brain activity relating to interoception (Cerritelli et al., 2020b). Nonetheless, future research is necessary to investigate if osteopathic treatment for patients with chronic pain and comorbid depression influences physical and mental symptoms and interoceptive, autonomic, and allostatic measures.

## Discussion and Future Directions

Taken together, we have proposed an embodied, predictive and interoceptive framework to reason and research the putative effect of osteopathic treatment on individuals suffering from persistent physical symptoms and comorbid mental health symptoms and disorders. The theoretical framework is based on up-to-date psychological and neuroscientific research and provides testable hypotheses. In other words, it is built on theoretical grounds and requires experimental scrutiny for verification or falsification. To this end, it seems expedient to investigate if osteopathic treatment applied to patients with, e.g., chronic pain and comorbid depression effectively increases interoceptive accuracy, decreases allostatic load, modulates autonomic nervous system activity, and alleviates physical and mental health symptoms using a range of research strategies such as clinical trials. Likewise, the putative effect of osteopathic treatment on brain functioning may be evaluated in this patient population using neuroimaging techniques with an emphasis on interoceptive and emotional brain networks (possibly using dynamic causal modeling). We have hypothesized that osteopathic treatment may increase interoceptive accuracy and benefit patients with physical and mental health symptoms and conditions that are upheld by overweighting of priors and low interoceptive accuracy. However, from a conceptual point of view, it is uncertain whether symptoms and disorders associated with overweighting of the likelihood and high interoceptive accuracy may also benefit from osteopathic treatment. If the perception of interoceptive information is fostered in treatment, it may be the case that symptom perception is increased. However, it is more likely that the interoceptive input provided during osteopathic treatment is distinct from the interoceptive information that maintains symptom perception, thus, leading to a reduction of symptoms. Still, both theoretical considerations need to be tested experimentally using interoceptive and symptom-specific measures to draw any conclusion.

To our knowledge, this framework is the first to apply active inference and predictive coding to osteopathic care for individuals with persistent physical symptoms and mental health comorbidities. However, it is worth noting that chronic pain and depression are highly complex symptoms and conditions that cannot be reduced to a few factors, either theoretically or practically. Similarly, osteopathy is a complex therapeutic approach that incorporates touch and manual therapy and patient management strategies, all anchored in a solid therapeutic alliance. Thus, this framework is constrained because it emphasizes touch-based mechanisms and largely overlooks the importance of the patient-therapist relationship. Furthermore, the critical role of environmental and sociocultural factors in developing these physical and mental health conditions is overlooked. Thus, this theoretical framework capture just a part of the person-centered nature of osteopathic care or the complexity of persistent physical and mental conditions; however, it should be understood in this context, and future research may consider and conceptually integrate these perspectives in order to advance the framework beyond mere touch. Additionally, it is worth noting that the current framework may be extended to other physical and manual therapies that employ body- and touch-based approaches in treating patients with physical and co-occurring mental disorders. This supports the framework’s overarching goal of fostering the development of a long-overdue collaborative approach between physical and mental health care specialists in the management of complex comorbidity health conditions.

**FIGURE 3:**
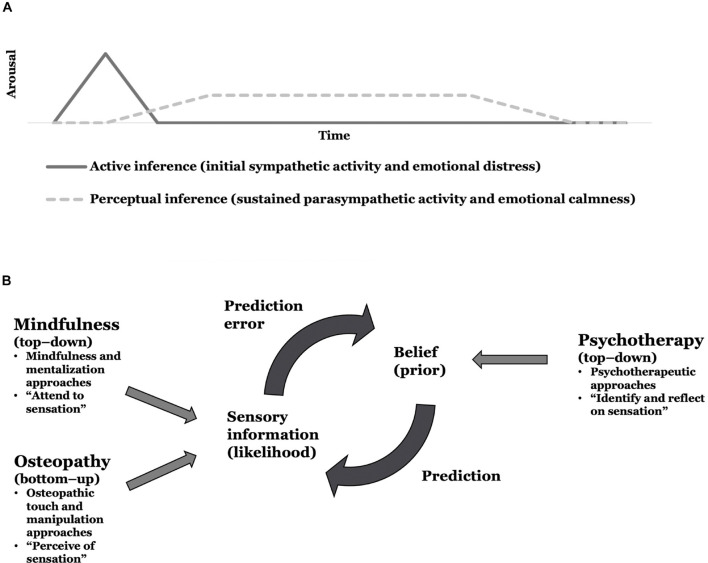
Implications for future research testing the theoretical framework. **(A)** An active and perceptual inference perspective to reason autonomic and emotional responses in osteopathic practice. **(B)** A proposition for a multidisciplinary interoceptive exposure therapy to physical and comorbid mental symptoms or conditions.

In the future, this theoretical framework might be used to reinterpret a phenomenon frequently encountered in osteopathic practice, namely, when manual treatment of peripheral tissues leads to autonomic and emotional responses (Upledger, 2002; Myers, 2014). From experience, these situations seem to involve initial sympathetic activity and emotional distress followed by sustained parasympathetic activity and emotional calmness. We argue that this phenomenon may evolve through active and perceptual inference processes that lead to a cascade of autonomic and emotional responses. Arguably, touch applied to a bodily region linked to physical and mental symptoms may produce interoceptive information that contradicts the predicted physical and mental states associated with e.g., pain and depression. This mismatch produces interoceptive prediction errors that are minimized using active and perceptual inference. It might be the case that an initial minimization using active inference engages the autonomic nervous system to produce the expected unpleasant sensation, thus leading to the initial distressing autonomic and emotional responses encountered in clinical practice; note that interoceptive prediction errors are used to infer emotional states (Allen, 2020). However, as the contextual factors in a healthcare setting do not promote such an inference, pending a safe and trusting therapeutic alliance is established, we suggest that perceptual inference processes are engaged to update the belief that predicts the unpleasant sensation with actual interoceptive information, thus, leading to the sustained pleasant autonomic and emotional responses (Figure 3A). In our experience, these autonomic and emotional responses seem to benefit psychological factors in patients with physical conditions. Although speculative, active and perceptual inference processes may underlie this phenomenon. However, clinical research using autonomic and emotional measures (e.g., heart-rate variability and fascial thermography) needs to be conducted to explore further this phenomenon’s underlying mechanisms and putative health benefits.

Furthermore, our framework provides a theoretical grounding on which to assess multidisciplinary collaborations between mental and physical healthcare specialists to treat comorbid physical and mental symptoms or health conditions. In detail, we propose an integrative interoceptive exposure therapy that may underpin multidisciplinary person-centered care. Therein, top-down mindfulness-based psychotherapeutic interventions may be combined with bottom-up touch-based osteopathic interventions to enhance interoceptive processing and reduce physical and mental symptoms. More specifically, we propose combining modalities from psychotherapy, mindfulness, mentalization, and osteopathy to identify, attend to, perceive, and reflect on bodily (interoceptive) sensations linked to physical and mental symptoms. Arguably, psychotherapeutic approaches may be used to identify and reflect on (or make sense of) interoceptive sensations (top-down), and mindfulness and mentalization approaches could be utilized to (actively and non-judgmentally) attend to interoceptive sensations (top-down), while osteopathic approaches might be applied to perceive (or produce) interoceptive sensations (bottom-up) in bodily regions that are linked to the physical and mental symptoms. Thus, both body- and mind-based treatment approaches could be combined in multidisciplinary collaborations to update the precision weighting between actual and expected interoceptive information. We hypothesize that bottom-up osteopathic, and top-down mindfulness and mentalization approaches may increase the precision afforded to the sensory information (likelihood), while top-down psychotherapeutic approaches may decrease the precision afforded to the belief (prior) (Figure 3B); thus, adjusting the weighting between expected and actual interoceptive information (in favor of the latter) as a means of updating the maladaptive belief issuing the prediction of pain and depression. However, whether osteopathic approaches may enrich psychotherapeutic and mindfulness approaches in treating persistent physical and mental symptoms and conditions needs to be formally tested in clinical research.

Taken together, this theoretical framework is based on contemporary theories from psychology and neuroscience but requires experimental scrutiny for validation or falsification. Nonetheless, it may inform future research addressing physical and comorbid mental symptoms and conditions in osteopathic practice; for example, assessing the phenomenon of autonomic and emotional responses to osteopathic touch and implementing multidisciplinary collaborations between mental and physical health specialists.

## Conclusion

This hypothesis and theory article introduced an embodied, predictive, and interoceptive framework to osteopathy and mental health. Based on research from embodied cognition, predictive coding, interoception, and osteopathy, this theoretical framework aims to provide a foundation to reason and research the effect osteopathy putatively has on comorbid psychological factors in patients with physical conditions. Osteopaths frequently treat patients with, for example, chronic pain and comorbid depression which may arguably be linked to false inferences and interoceptive deficits including overly precise interoceptive predictions (e.g., expecting interoceptive information linked to physical and mental states associated with pain and depression) and imprecise interoceptive prediction errors (e.g., not sensing interoceptive information linked to physical and mental states not associated with pain and depression). Osteopathic manual therapeutic approaches may aim to provide uncertain and surprising interoceptive information (being contrary to the predicted information) to the bodily area that is associated with the patients’ physical and comorbid mental symptoms to update these maladaptive interoceptive predictions and improve the ability to attend to interoceptive information. Notably, these manual techniques need to be complemented by patient management approaches involving reassurance, education, support, advice, and exercises to provide cognitive grounding for the revision of the generative model responsible for the interoceptive predictions and attenuation to interoceptive information, and integrate these changes into everyday life. In that, osteopathic interventions arguably generate interoceptive prediction errors (mismatch between expected and actual interoceptive information) that are minimized using active and perceptual inference processes. To this end, osteopathic care plays a crucial role in allostatic regulation and therefore health and wellbeing, particularly through active interoceptive inference. We have suggested that during osteopathic treatment, first, active inference processes may be engaged that lead to autonomic activity, which resembles the predicted physical and mental symptoms associated with pain and depression—as these are inferred to be the likely cause of uncertain interoceptive information. However, as the interoceptive input emerges in a healthcare setting, perceptual inference processes may be engaged to update the prediction and underlying belief according to actual interoceptive information, while also improving the ability to attend to interoceptive information. In a nutshell, persistent and noisy interoceptive prediction errors (arguably maintaining symptoms) may putatively be “replaced” with surprising and precise interoceptive prediction errors (arguably alleviating symptoms). In this way, osteopathic treatment might reduce the belief about, and prediction of, physical and mental states associated with chronic pain and comorbid depression.

## Data Availability Statement

The original contributions presented in the study are included in the article/supplementary material, further inquiries can be directed to the corresponding author/s.

## Author Contributions

LB wrote the first draft of the manuscript. All authors contributed to the conception of the manuscript, contributed to manuscript revision, approved, and were accountable for the submitted manuscript.

## Conflict of Interest

FC and JE were Topic Editors for the Research Topic: “Enactivism and Active Inference in the Therapeutic Alliance” but were not involved in the review or approval of this manuscript. The remaining authors declare that the research was conducted in the absence of any commercial or financial relationships that could be construed as a potential conflict of interest.

## Publisher’s Note

All claims expressed in this article are solely those of the authors and do not necessarily represent those of their affiliated organizations, or those of the publisher, the editors and the reviewers. Any product that may be evaluated in this article, or claim that may be made by its manufacturer, is not guaranteed or endorsed by the publisher.

## Footnotes

1 Persistent physical symptoms (also referred to as medically unexplained symptoms or functional motor and sensory symptoms) are characterized by somatic symptoms (e.g., pain and fatigue) with a chronic representation (>6 months) that cause sufficient suffering.

2 It is notable that the osteopathic profession did not prioritize research in the past and that currently steps are being taken to build an osteopathic research culture (Beverly, 2021), which is reflected by the recent establishment of research networks (Steel et al., 2020b) and the exponential growth in publication output during the past decades (Morin and Gaboury, 2021).

3 Although osteopathy should solely be utilized in the treatment of mental disorders if applied adjuvant to specialist mental health service, it is noticeable that mental disorders and osteopathy show putative overlap in the way the organism is affected, which may inform future research. For example, mental disorders seem to be associated with (1) autonomic (Alvares et al., 2016), (2) inflammatory (Savitz and Harrison, 2018), (3) interoceptive (Khalsa et al., 2018) as well as (4) homeostatic and allostatic changes (Sterling, 2014; Carbone, 2021), whereas osteopathy was indicated to (1) regulate autonomic nervous system activity (Ruffini et al., 2015; Rechberger et al., 2019; Cerritelli et al., 2020a), (2) produce anti-inflammatory effects (D’Alessandro et al., 2016) both *in vitro* (Zein-Hammoud and Standley, 2015; Anloague et al., 2020) and *in vivo* (Licciardone et al., 2012), (3) affect interoceptive processing and its neural correlates (Cerritelli et al., 2020b), and (4) aims to enhance homeostasis functioning (Bergna et al., 2020) and reduce allostatic load (Lunghi et al., 2020).

4 “Maladaptive” is a term commonly used in the neuroscience and active inference literature, but the authors are aware there can be some misinterpretation of this term. It is used here for clarification purposes and not to privilege the practitioner’s discourse over the patient’s narrative.
